# A four-dimensional snapshot hyperspectral video-endoscope for bio-imaging applications

**DOI:** 10.1038/srep24044

**Published:** 2016-04-05

**Authors:** Hoong-Ta Lim, Vadakke Matham Murukeshan

**Affiliations:** 1 Centre for Optical and Laser Engineering, School of Mechanical and Aerospace Engineering, Nanyang Technological University, 50 Nanyang Avenue, 639798 Singapore

## Abstract

Hyperspectral imaging has proven significance in bio-imaging applications and it has the ability to capture up to several hundred images of different wavelengths offering relevant spectral signatures. To use hyperspectral imaging for *in vivo* monitoring and diagnosis of the internal body cavities, a snapshot hyperspectral video-endoscope is required. However, such reported systems provide only about 50 wavelengths. We have developed a four-dimensional snapshot hyperspectral video-endoscope with a spectral range of 400–1000 nm, which can detect 756 wavelengths for imaging, significantly more than such systems. Capturing the three-dimensional datacube sequentially gives the fourth dimension. All these are achieved through a flexible two-dimensional to one-dimensional fiber bundle. The potential of this custom designed and fabricated compact biomedical probe is demonstrated by imaging phantom tissue samples in reflectance and fluorescence imaging modalities. It is envisaged that this novel concept and developed probe will contribute significantly towards diagnostic *in vivo* biomedical imaging in the near future.

Hyperspectral imaging (HSI) can collect required spectral information from each spatial pixel in the region of interest (ROI). By arranging the spectral information according to the positions, a three-dimensional (3-D) datacube can be realized with the spatial information in two dimensions and the spectral information arranged in the third dimension^1^. Each voxel gives the optical information of one wavelength from one spatial position^2^. Typically, in order to be defined as a hyperspectral (HS) imager, the resulting datacube should have at least 100–200 images at different wavelengths^3^. Compared to multispectral imaging which captures at most few tens of wavelength bands^3^, HSI captures much more wavelengths and thus the spectral gap between adjacent wavelengths reduces and the spectral signatures are more detailed. HSI has mainly three reported configurations which are named as spatial-scanning, spectral-scanning and snapshot methods. They differ in how the 3-D data is acquired to form the 3-D datacube. This leads to each method having a set of characteristics which makes it only suitable for certain applications.

A spatial-scanning HSI system can be a whiskbroom or a pushbroom type and usually uses a dispersive element to separate the incoming spectrum^4^. In each scan, a whiskbroom HSI system acquires the one-dimensional (1-D) spectral information of one spatial point of the ROI at a time^5^, using its 1-D single-line detector. After repeating the scan over multiple points over the area of the ROI, the 1-D spectra are arranged according to where they were collected to form the datacube. A pushbroom HSI system has a narrow slit allowing a straight line of light to pass through the slit^6^. After spectral dispersion, a two-dimensional (2-D) spatial-spectral data is collected by the 2-D detector in each scan. Scanning is repeated after a relative displacement between the sample and the imager, in the direction transverse to the slit, so that the next line can be imaged. After the entire ROI is imaged, the arrangement of the multiple 2-D data according to the sequence in which they were collected forms the datacube. A spatial-scanning imager typically acquires many images from hundreds of wavelengths, giving detailed spectral signatures across a large spectral range. However, the scanning takes a longer time to complete when a larger ROI is imaged. Sequential scanning is required by both whiskbroom and pushbroom imagers. However, the pushbroom configuration is more efficient than the whiskbroom method, as it collects spectral information from a larger area of the sample in each scan^7^.

A spectral-scanning HS imager uses a tunable filter whose spectral transmission is controlled electronically^8^. It allows light close to a selected wavelength to be transmitted at a time, which is later detected by the 2-D detector placed behind the tunable filter. Liquid crystal and acousto-optic tunable filters have been used in such HSI systems^9^,^10^. Each captured image is a 2-D spatial-spatial data of a narrow wavelength band. Repeated scanning is performed after the tunable filter switches the wavelengths to be transmitted. By arranging the multiple 2-D data according to the transmitted wavelength of the tunable filter, a datacube is formed. Sequential scanning is required for switching the transmitted wavelength of the tunable filter, and the total acquisition time is longer when the number of wavelengths to interrogate becomes larger.

Snapshot HSI systems are able to capture the 3-D data to build a datacube in a single scan^11^. This is done using different configurations such as integral-field spectroscopy^12^, image mapping spectroscopy^13^,^14^, computed tomographic imaging spectroscopy^15^ and compressive sensing^2^. Such systems do not need sequential scanning to build a datacube. The ability of the snapshot imager to capture the 3-D data in one scan has both advantages and limitations. The main benefit of such an imager is that it is much faster than spatial- and spectral-scanning HSI systems, and can be used in real-time applications depending on the exposure time and the detector’s readout rate. Motion artifacts and pixel misregistration can therefore be eliminated^15^. Each 2-D detector has a limited number of pixels and can only capture that much information in one scan. Thus snapshot imager can only acquire a limited amount of information, and has to sacrifice on the number of spatial points or wavelengths from which the data is collected.

HSI was first used in airborne and spaceborne vehicles for the observation of Earth^5^. Its ability to capture data to form a datacube consisting of hundreds of images from contiguous and narrow spectral bands for further analysis has since led to many other applications. These include astronomy^16^,^17^, examination of historical murals^18^, quality assessment of food^19^, and bio-imaging^20^,^21^,^22^. In biomedical imaging, endoscopes have been developed to image sites within the body that are not easily accessible by conventional table-top setup. HSI has been incorporated into such applications using the spectral-scanning^23^,^24^ and snapshot methods^13^. In real-time endoscopic applications such as *in vivo* disease diagnosis and surgical monitoring, the snapshot methods will be the better choice. However, one major drawback of existing snapshot HS endoscopes is that only ~50 spectral bands can be acquired^13^.

In this context, we have developed a snapshot HS probe which can be used for endoscopic diagnostics biomedical applications. The custom fabricated probe is flexible along its length and its distal end has a small profile so that it can be inserted into the orifice of the body cavities such as the anus to investigate the gastrointestinal tract. It is a 2-D array (10 × 10) of hexagonally-packed optical fiberlets (individual fibers) arranged in in rows and columns, which are orderly rearranged to form a 1-D array (1 × 100) of fiberlets at the other end. This arrangement allows the 2-D image to be captured by 2-D end of the fiber bundle and be reduced from two spatial dimensions to only one dimension at its 1-D end^25^. The light from the 1-D end of the fiber bundle enters a spectrograph which disperses the light to be detected by the 2-D detector. This 2-D to 1-D fiber bundle reduces the 3-D data (spatial-spatial-spectral) to a 2-D data (spatial-spectral) so that the 2-D detector takes only one scan to capture all the information. Capturing the data sequentially in real-time adds a fourth dimension to the data collected, which is in the temporal domain^26^. After data acquisition, custom written software was used to process and rearrange each spectrum acquired in a scan according to the spatial position from which it was acquired on the 2-D end of the fiber bundle. It is to be mentioned that though the use of 2-D to 1-D fiber bundle has been previously reported, it was only used as an optical element in a field or table-top systems^26^,^27^,^28^. These systems using 2-D to 1-D fiber bundles do not have a flexible, long and small probe.

In this paper, the use of a custom fabricated flexible 2-D to 1-D fiber bundle as a compact four-dimensional (4-D) snapshot HS video-endoscope is illustrated for diagnostic biomedical applications. It forms an image of the sample covering ~1.11 × 1.32 mm^2^ of 100 spatial points at a frame rate of ~6.16 Hz. The spectral range of interest is 400–1000 nm with 756 spectral bands.

## Results

### Reflectance imaging

**Figure 1:**
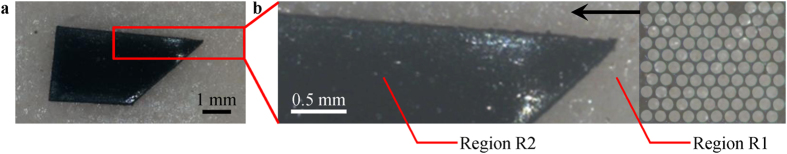
(**a**) Simulated phantom tissue sample for reflectance imaging and (**b**) the photograph of the 2-D end of the fiber bundle superimposed on the imaged region of the sample. Region R1 represents normal tissue while Region R2 represents a tumor located on the tissue surface with different reflectance properties. The fiber bundle in (**b**) shows its initial position and the arrow indicates its relative movement with respective to the sample during data acquisition.

The tissue properties of normal tissue and tumor are different and the possible mechanisms include mucosal thickening and higher vasculature density in abnormal lesions^29^. These can result in the optical reflectance between a normal tissue and tumor to be different, especially when the tumor is located on the tissue surface. In order to simulate this situation of normal and abnormal tissue regions, a black tape was placed on a phantom tissue sample (Fig. 1a) and imaged while being illuminated by two flexible light guides which delivers light from a broadband white light source. The sample is divided into Regions R1 and R2. Region R1 is the phantom tissue sample representing normal tissue, while Region R2 is the black tape representing a tumor located on the tissue surface with different reflectance properties^29^,^30^. The sample was manually moved using a mechanical stage towards the right of the 2-D end of the fiber bundle during data acquisition. With respect to the sample, the 2-D end of the fiber bundle was moving to the left (arrow in Fig. 1b). A total of 80 frames were taken at a rate of ~6.16 Hz.

**Figure 2:**
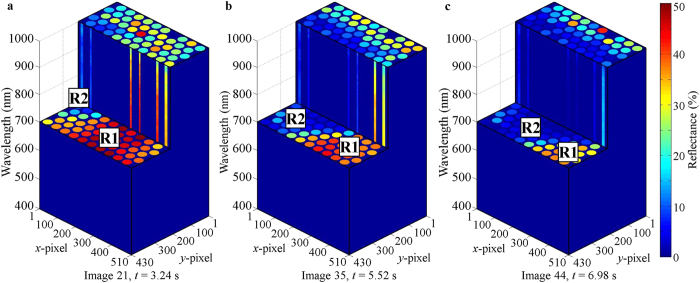
Each frame captured by the detector camera was used to build a 3-D datacube. (**a–c**) show the cut-datacube acquired using frames 21, 35 and 44, which were captured ~3.24 s, 5.52 s and 6.98 s respectively after data acquisition started. These 3-D datacubes show the 4-D data (spatial-spatial-spectral-temporal) captured using the snapshot HS video-endoscope. It can be observed that with respect to the sample, the 2-D end of the fiber bundle was moving towards the left during data acquisition.

Each frame captured by the detector camera of the snapshot HS video-endoscope was used to build a 3-D datacube. The datacubes were built at the same rate as the frame rate of the detector camera ~6.16 Hz. 80 frames were captured in ~12.81 s. Three cut-datacubes are shown in Fig. 2. These 3-D datacubes show the 4-D data (spatial-spatial-spectral-temporal) captured using the snapshot HS video-endoscope.

**Figure 3:**
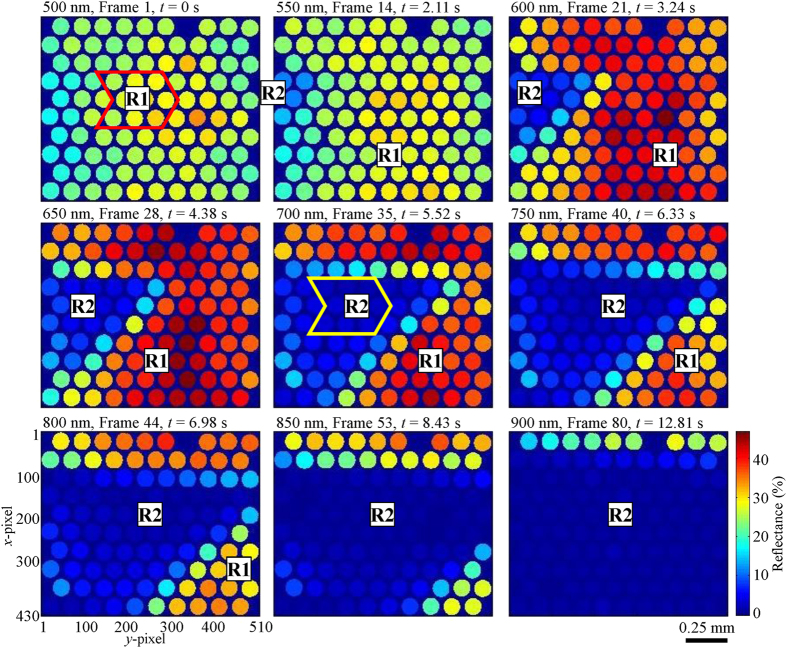
4-D reflectance mappings of nine selected wavelengths and datacubes taken at different timings. The 2-D end of the fiber bundle was moving towards the left with respect to the sample during data acquisition.

Figure 3 shows the reflectance mappings of nine wavelengths and datacubes. Supplementary Videos 1 and 2 are made up of 80 frames each, using reflectance mappings of 575 nm and 700 nm respectively. By looking at the frames in Figs 2 and 3 sequentially and Supplementary Videos 1 and 2, it can be observed that the proposed system was able to perform HS reflectance imaging in a snapshot configuration. The different reflectance between Regions R1 and R2 was captured and they can be differentiated from each another. The 2-D end of the fiber bundle was initially imaging Region R1 of high reflectance, representing normal tissue region. Then it moved to the left with respect to the sample and entered Region R2 of low reflectance, representing the abnormal region of the tissue. The sharp tip on the right of Region R2 can be clearly imaged. This continued in the same direction while imaging Region R2 till the data acquisition stopped. These depict the actual relative motion between them during data acquisition (Fig. 1b). The shape of Region R2 is also correctly represented in the experimental results.

**Figure 4:**
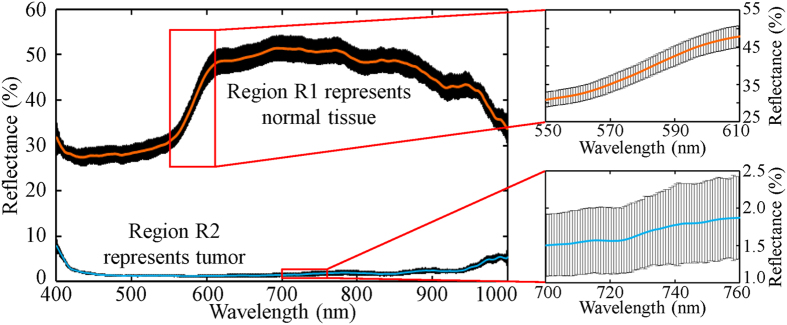
Reflectance spectra of Regions R1 and R2. Mean reflectance spectrum (center bold line) and standard deviation (surrounding black areas) along with insets.

The mean reflectance spectra and standard deviations of Regions R1 and R2 are shown in Fig. 4. Each data set was calculated from 27 spectra. The spectra of Region R1 were acquired from 9 fiberlets whose positions are indicated by the red arrow box in the intensity mapping shown for 500 nm (see Fig. 3) and from Frame 3–5. The spectra of Region R2 were acquired from 9 fiberlets whose positions are indicated by the yellow arrow box in the intensity mapping for 700 nm as shown in Fig. 3 and from Frame 35–37.

The spectra in Fig. 4 show that the 4-D HS imaging probe could capture the detailed reflectance spectra of Regions R1 and R2 while there was a relative motion between the sample and the 2-D end of the fiber bundle. It can be observed that Region R1 (phantom tissue sample) had a much higher reflectance than Region R2 (black tape) along the entire spectral range of interest. The average standard deviations of the reflectance spectra of Regions R1 and R2 were about ±2.49% and ±0.42% respectively.

The results in this section show that 4-D HS imaging probe was able to capture the HS reflectance of different parts of the imaged sample throughout the duration of data acquisition. Reflectance intensity mappings of appropriate wavelengths can be selected to spectrally distinguish one region from another for diagnostic applications. The spectral information collected from known samples can be stored in a data library and be used for identification and quantification in future. The capabilities of the proposed imaging probe are also demonstrated in another experiment conducted on a chicken breast tissue with blood clot. The results are detailed in the Supplementary Discussion (Supplementary Figs 1–3 and Supplementary Videos 3 and 4).

### Fluorescence imaging

**Figure 5:**
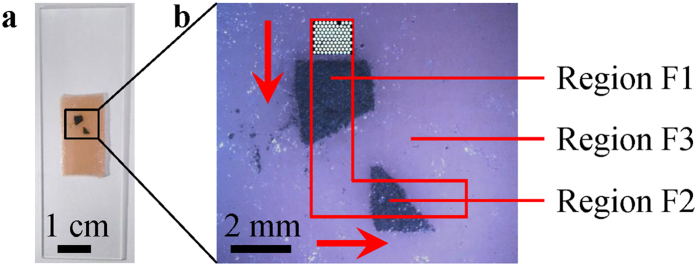
(**a**) Simulated phantom tissue sample for fluorescence imaging and (**b**) the photograph of the 2-D end of the fiber bundle superimposed on the sample. Region F1 represents normal tissue with normal autofluorescence intensity. Region F2 represents tumor growth in the intermediate stage with reduced autofluorescence intensity. Region F3 represents tumor growth in the advanced stage with very weak autofluorescence. The 2-D end of the fiber bundle was initially placed on the top of the marked area. The arrows in (**b**) indicate its relative movement with respect to the sample during data acquisition. (**a**,**b**) are images of the same sample but appear to have different colors due to the different illuminations and cameras used.

Endogenous fluorophores such as flavin, collagen and porphyrins^29^,^31^,^32^ are natural fluorophores. Autofluorescence occurs when these endogenous fluorophores are excited by a laser having an appropriate excitation wavelength. The differences in the tissue microarchitecture and concentration of endogenous fluorophore between a normal tissue and tumor^29^ can lead to variations in autofluorescence^29^,^31^,^33^. Depending on the excitation wavelength, autofluorescence in tumor can be lower^29^,^33^ or higher^33^ compared to normal tissues. This is due to the excited endogenous fluorophores having lower or higher concentrations in tumor respectively^33^. The magnitude of autofluorescence variations is dependent on the tumor stage since the differences become greater as the tumor progresses. Therefore, detection and staging of tumor can be done using fluorescence imaging to capture the autofluorescence in tissues by looking out for changes in the fluorescence intensity. In this experiment, a phantom tissue sample with applications of fluorescent powder (Fig. 5a), simulating different stages of caner growth in colon, was imaged while being illuminated by two flexible light guides delivering light from a 532-nm laser. The fluorescent powder has emission wavelengths from ~500–700 nm, and falls within the emission maxima of biological endogenous fluorophores (280–690 nm)^32^. The sample is divided into Regions F1, F2 and F3. Region F1 is simulated with a thick region of fluorescent powder (higher concentration) representing normal tissue having normal autofluorescence intensity. Region F2 is simulated with a thin region of fluorescent powder (lower concentration) representing tumor growth in the intermediate stage with reduced autofluorescence intensity. Region F3 is the simulated phantom tissue sample representing tumor growth in the advanced stage with very weak autofluorescence. The sample was manually moved using a mechanical stage first upwards, then towards the left of the 2-D end of the fiber bundle during data acquisition. With respect to the sample, the 2-D end of the fiber bundle was first moving downwards then to the right (arrows in Fig. 5b). A total of 160 frames were taken at a rate of ~6.16 Hz.

**Figure 6:**
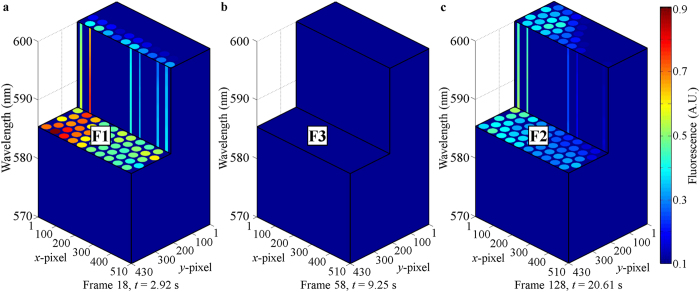
Each frame captured by the detector camera was used to build a 3-D datacube. (**a–c**) show the cut-datacube acquired using frames 18, 58 and 128, which were captured ~2.92 s, 9.25 s and 20.61 s respectively after data acquisition started. (**a**) was acquired from Region F1 representing normal tissue with detection of high fluorescence intensity. (**b**) was acquired from Region F3 representing tumor growth in the advanced stage with detection of very low fluorescence intensity. (**c**) was acquired from Region F2 representing tumor growth in the intermediate stage with detection of low fluorescence intensity. These 3-D datacubes show the 4-D data (spatial-spatial-spectral-temporal) captured using the snapshot HS video-endoscope.

In this experiment, the snapshot hyperspectral video imager captured 160 frames in ~25.79 s. Each frame was used to build a 3-D datacube. Although the full spectral range of 400–1000 nm was captured, only the data from 570–600 nm is used to build the three cut-datacubes shown in Fig. 6. Figure 6a representing Region F1 indicates normal tissue with detection of high fluorescence intensity. Figure 6b representing Region F3 indicates tumor growth in the advanced stage with detection of very low fluorescence intensity. Figure 6c was acquired from Region F2 which represents tumor growth in the intermediate stage with detection of low fluorescence intensity. These 3-D datacubes show the 4-D data (spatial-spatial-spectral-temporal) captured using the snapshot HS video-endoscope.

**Figure 7:**
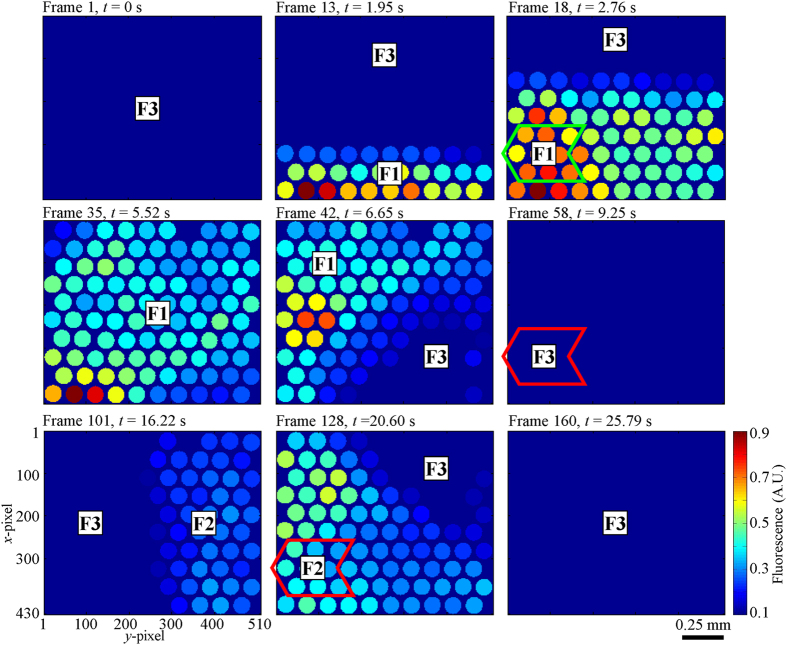
Fluorescence mappings of nine datacubes at 585 nm taken at different timings. With respect to the sample, the 2-D end of the fiber bundle was moving downwards before moving towards the right during data acquisition.

Figure 7 shows the fluorescence mappings of nine datacubes at 585 nm. Supplementary Videos 5 and 6 are made up of 125 frames each (Frame 11–135), using fluorescence mappings of 575 nm and 585 nm respectively. By looking at the frames in Fig. 7 sequentially and Supplementary Videos 5 and 6, it can be observed that the proposed snapshot system can be used to perform HS fluorescence imaging. The fluorescence intensities of Regions F1, F2 and F3 were captured and the information can be used to differentiate one from another. The 2-D end of the fiber bundle was initially imaging Region F3 of very low fluorescence intensity. It moved downwards with respect to the sample and entered Region F1 of high fluorescence intensity. Then it entered Region F3 before moving towards the right with respect to the sample and entered Region F2 of low fluorescence intensity. It continued in this path until it entered Region F3 before data acquisition stopped.

These depict the actual relative motion between them during data acquisition (Fig. 5b). It is to be noted that there was uneven illumination on the phantom sample where the illumination on the left was stronger. The shapes of Regions F1 and F2 are also correctly represented in the experimental results.

**Figure 8:**
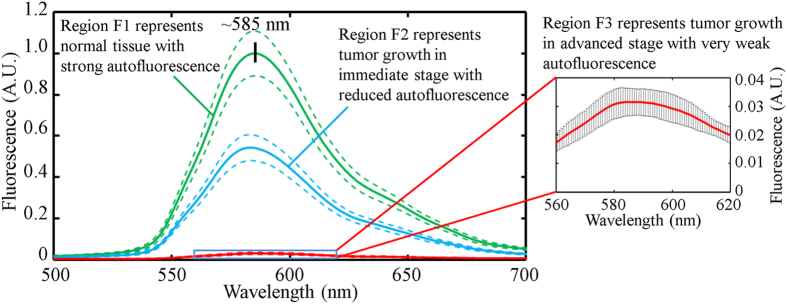
Fluorescence spectrum of Regions F1, F2 and F3. Mean fluorescence spectrum (center bold line) and standard deviation (dotted lines) with inset of Region F3.

The mean fluorescence spectra (solid lines) and standard deviations (dotted lines) of Regions F1, F2 and F3 are shown in Fig. 8. Each data set was calculated from 27 spectra and normalized to the maximum value of the mean fluorescence spectrum of Region F1. The spectra were acquired from the same 9 fiberlets whose positions are indicated by the green and red arrow boxes in Fig. 7. For the purpose of comparing the spectral intensity of Regions F1, F2 and F3, the effect of uneven illumination on the sample is reduced by acquiring the spectra from the same fiberlets. The spectra of Regions F1, F2 and F3 were acquired from Frame 17–19, 127–129 and 57–59 respectively.

The spectra in Fig. 8 illustrate that the 4-D HS imaging probe could capture the detailed fluorescence spectra of Regions F1, F2 and F3 while there was a relative motion between the sample and the 2-D end of the fiber bundle. The peak fluorescence wavelength was ~585 nm. It can be observed that strong fluorescence was detected from Region F1 (thick fluorescent powder region with higher concentration) representing normal tissue with normal autofluorescence intensity. A relatively weaker fluorescence was detected from Region F2 (thin fluorescent powder region with lower concentration) representing tumor growth in the intermediate stage with reduced autofluorescence intensity. A very weak fluorescence was detected from Region F3 (phantom tissue sample) representing tumor growth in the advanced stage with very weak autofluorescence. The average standard deviations of the fluorescence spectra of Regions F1, F2 and F3 from 500–700 nm are about ±0.0368, ±0.0213 and ±0.0026 respectively (Fig. 8).

The results in this section show that 4-D HS imaging probe captured the HS fluorescence of different parts of the imaged sample throughout the duration of data acquisition. The system can capture the fluorescence spectra which can reveal the type and concentration of fluorophores in the samples. This can in turn leads to tumor staging and other related disease diagnostics applications.

## Discussion

Based on the technical specifications of an existing pushbroom HS imager, we have a custom-fabricated 2-D to 1-D fiber bundle that converts the pushbroom HS imager into a snapshot configuration. The fiber bundle is also flexible and has a small distal end enabling it to be used as an imaging probe that can be inserted into the body for minimally invasive and *in vivo* investigations. By acquiring data frames continuously, these factors come together to give a snapshot HS video-endoscope with potential bio-imaging applications.

We have given a detailed instrumentation of the proposed system and demonstrated its feasibility. Reflectance and fluorescence imaging were conducted when the light source and the probe were both on the same side as that of the imaged samples. This is the expected configuration during *in vivo* imaging of the internal body cavity. Further, it is to be noted that this probe can be integrated with a control and locomotion option as in conventional endoscopes avoiding the need for sample movement when this is to be used inside body cavities.

In both reflectance and fluorescence imaging, we were able to attain a frame rate of ~6.16 Hz, and each frame was converted into a 3-D datacube with 756 spectral bands. The 3-D datacubes and intensity mappings can provide vast amount of information, which includes the spatial features (size and shape), spectral signatures (756 bands), speed and direction of the imaged samples. The spectral information can also be seen in the line plots. These promising results confirm the successful implementation of such a 2-D to 1-D fiber bundle serving for its use as a snapshot HS video-endoscopic probe. The snapshot HS video-endoscope illustrated in this paper, used the flexible 2-D to 1-D fiber bundle for potential bio-imaging applications for the first time. It also captures 756 spectral bands which are significantly more than existing snapshot HS video-endoscopes which can generally capture only ~50 spectral bands. With more spectral bands available, limitations such as a reduced spectral range, insensitivity to certain narrow spectral band and inability to capture detailed spectral signatures, can be avoided.

The use of such a HS video-endoscope with a flexible 2-D to 1-D fiber bundle can be a potential alternative to conventional fiber optic imaging systems. The information collected by a HS video-endoscope has an additional spectral dimension of the order of several hundred wavelength bands. Conventional video-endoscope using color camera gives only little spectral information from three bands. In this aspect, HS video-endoscopes can be especially useful when detailed spectrum is required for classification and quantification to give functional information such as hemoglobin saturation^9^. It is also valuable in cases where multiple excitation sources are used to excite multiple fluorescent tags and the HS data can be used to differentiate the fluorescent tags even when the excitation and emission spectra are over-lapping but distinct^34^. Currently, many conventional setups in the field of optogenetics and neuronal imaging can only image one fluorescent tag in each frame^35^,^36^,^37^. Such studies can benefit by using HS video-endoscope with a flexible and compact distal end. It can be used in more complex and non-invasive studies to capture detailed spectral information from multiple fluorescent tags.

A future improvement to the probe system is to use smaller fiberlets so that more can be packed within the fiber bundle. The current system images 100 fiberlets on the 1-D end using all the 1004 pixel columns. While the maximum number of fiberlets that can be effectively imaged by the snapshot imager is 1004, which is the number of pixel column of the sensor array. By using smaller fiberlets, more can be packed along the 1-D end. Spectral information from more spatial points will be collected and the data collection by the sensor array becomes more efficient. The image quality in terms of spatial dimensions is also expected to be better. Another possible improvement to the system is to increase its frame rate from the current ~ 6.16 Hz to 20 Hz so that it can be real-time. The current frame rate is limited by the detector camera and exposure time. This can be made a complete real-time system by replacing the detector camera with another one having faster readout rate and by using a lower exposure time.

## Methods

### HS imaging system

**Figure 9:**
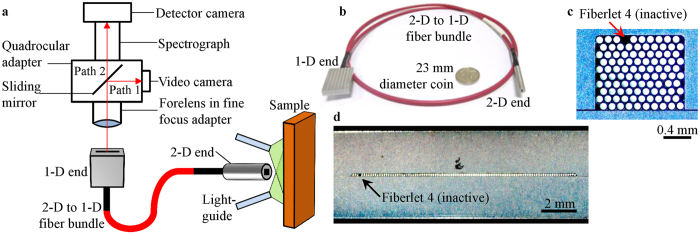
(**a**) Schematic diagram of HS imaging system, (**b**) photograph of 2-D to 1-D fiber bundle placed near a 23 mm diameter coin, (**c**) photograph of 2-D end-face showing all fiberlets and (**d**) photograph of 1-D end-face showing all fiberlets. 100 fiberlets were used in the fiber bundle, but only 99 fiberlets can be used for imaging (Fiberlet 4 was damaged and inactive).

The snapshot HS endoscopic probe shown in Fig. 9a was developed and installed in-house. This has two main parts, the HS imager and the 2-D to 1-D fiber bundle probe. The HS imager is the major part of a pushbroom HS imager which was previously reported by our group^20^, without the 3-axis motorized stage. During hardware installation, the video camera (UI-1550LE-C-HQ, iDS) and the detector camera (LucaEM DL-604M-OEM, Andor) had been positioned to produce focused images at the same time. The forelens was a doublet (2-50145, Navitar) kept in a fine focus adaptor (2-16265, Navitar). This adaptor was attached to bottom side of the quadrocular adaptor (Y-QT, Nikon), which housed a sliding mirror. The sliding mirror was pushed into the quadrocular adaptor and directed light towards the video camera (Path 1 in Fig. 9a). The video camera allowed direct video imaging and was used to position the 1-D end of the fiber bundle. With the 1-D end of the fiber bundle in place, the sliding mirror was pulled out of the quadrocular adaptor so that light can travel straight towards the spectrograph (ImSpectors V10E, Specim) and the detector camera (Path 2 in Fig. 9a). The spectrograph dispersed the light and the detector camera recorded the information required to build a datacube with each scan. A broadband light source (MI-150, Edmund Optics) and a 532-nm diode-pumped solid-state laser were used during reflectance and fluorescence imaging, respectively. The samples were illuminated using two flexible light-guides. During fluorescence imaging, a 550-nm long-pass filter (FEL0550, Thorlabs) was kept before the forelens. Spectral calibration was carried out and the probe system was found to be able to detect 756 spectral bands within a range of 400–1000 nm.

The 2-D to 1-D fiber bundle was custom fabricated by Polymicro Technologies^TM^ according to author’s specifications. The 100 optical fiberlets in the fiber bundle have core and buffer diameters of 100 μm and 125 μm, respectively (FVP100110125, Molex). They were arranged in a 10 × 10 hexagonally-packed fashion in the 2-D end and numbered from 1 to 100 across the column towards the right and then down the row (Fig. 9c). The vertical and horizontal core-to-core spacing on the 2-D end-face were ~110 μm and ~125 μm, respectively. The packing ratio on the 2-D end-face was ~55%. The fiberlets were then rearranged by row and column in a 1 × 100 fashion in the 1-D end and numbered correspondingly from 1 to 100 towards the right (Fig. 9d). The core-to-core spacing was ~125 μm. Fiberlet 4 was damaged and found to be inactive as indicated by the dark spot shown in Fig. 9c,d, thus cannot be used for imaging. The flexible bundle has a length of ~1 m with a 3.5 mm diameter polyvinyl chloride jacket. The optical fiberlets in the 1-D end were encased in a stainless steel holder with an end-face area of 5 × 20 mm^2^ and length of 30 mm. While in the 2-D end, they were enclosed in a cylindrical stainless steel holder with a diameter of 5 mm and length of 30 mm.

The full line-of-view of the HS imager was measured to have a length of ~12.5 mm. After selecting the optical fiberlet having a buffer diameter of 125 μm, it was estimated that the HS imager would only image ~100 optical fiberlets placed in a straight line. Hexagonally-packed optical fiberlets result in a bundle with a higher packing ratio compared to one which is packed in a square array. Therefore 100 optical fiberlets were used to make the fiber bundle and arranged in a hexagonally-packed fashion in the 2D-end.

### Spatial calibrations

**Figure 10:**
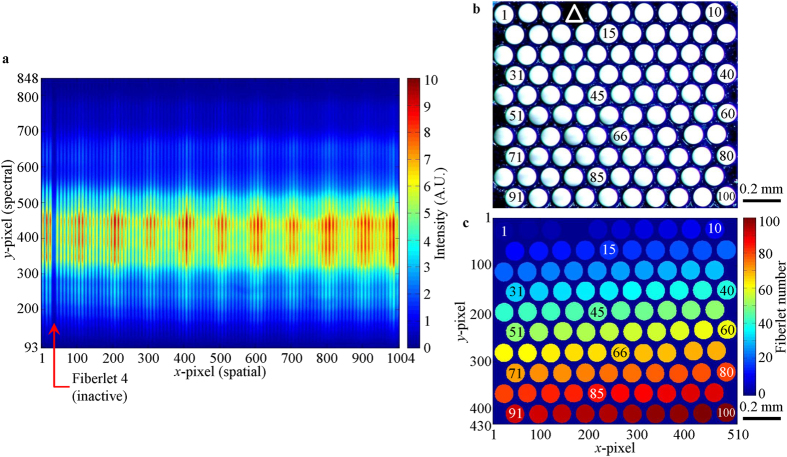
(**a**) Reference image taken by detector camera, (**b**) photograph of fiberlets on 2-D end-face and (**c**) digital mask of 2-D end-face. Both (**b**,**c**) are ~1.11 × 1.32 mm^2^. The numbers on some of the fiberlets in (**b**,**c**) indicate the numbering of the fiberlets. The white triangle in (**b**) indicates the position of Fiberlet 4 which was inactive and thus appeared to be dark.

The spatial calibration was conducted initially on the 1-D end of the fiber bundle after alignment and was fixed in place. The detector camera had a sensor array of 1002 rows (*y*-axis, spectral) and 1004 columns (*x*-axis, spatial), and it captured a 2-D spectral-spatial data (Fig. 10a) from the fiberlets in 1-D end of the fiber bundle. Each colored vertical line in Fig. 10a came from the light exiting the core of each fiberlet, which was then spectrally dispersed along the *y*-axis of the sensor array. The *y*-axis was later converted to the calibrated spectral bands^20^. The position of each colored vertical lines along the *x*-axis indicated the pixel columns used to image each fiberlet. Spectral information from each fiberlet would be acquired from the corresponding pixel columns during data processing. It can be observed from Fig. 10a that all the 1004 pixel columns of the sensor array was used to image the 100 fiberlets on the 1-D end. ~10 *x*-pixels were used to image each fiberlet. The dark line in Fig. 10a indicated the position of Fiberlet 4, which was damaged and thus inactive.

The second spatial calibration was done on the 2-D end of the fiber bundle. Figure 10b shows the photograph of all the fiberlets on the 2-D end-face which were illuminated from the other end of the fiber bundle. Using Fig. 10b, a digital mask of the 2-D end-face was created (Fig. 10c), which contained the position and numbering of some fiberlets. The spectrum acquired from each fiberlet in Fig. 10a will eventually be placed in the corresponding position in Fig. 10c.

### Sample preparation

In reflectance imaging, the sample used was a simulated tissue (obtained from Simulab Corporation) placed on a glass slide. This phantom tissue is a standard test sample used here for proof of concept studies. A black tape was then placed on the tissue phantom. A 99% reflectance standard (SRS-99-010, Labsphere) was also imaged for reflectance imaging. In fluorescence imaging, fluorescent powder (Ultra-orange/yellow fluorescent power, Medtech Forensics) was coated on the tissue phantom to simulate the different stages of tumor as illustrated in the manuscript. All the imaged objects were kept at a distance of ~0.5 mm away from the 2-D end-face of the fiber bundle. The samples were manually moved using high-resolution mechanical translational stages during imaging. Details regarding the data acquisition, processing and visualization are given in Supplementary Discussion.

## Additional Information

**How to cite this article**: Lim, H.-T. and Murukeshan, V. M. A four-dimensional snapshot hyperspectral video-endoscope for bio-imaging applications. *Sci. Rep.* **6**, 24044; doi: 10.1038/srep24044 (2016).

## Supplementary Material

Supplementary Information

Supplementary Video 1

Supplementary Video 2

Supplementary Video 3

Supplementary Video 4

Supplementary Video 5

Supplementary Video 6
