# Supplementary material for: A four-dimensional snapshot hyperspectral video-endoscope for bio-imaging applications
Source: Sci Rep. 2016 Apr 5;6:24044. doi: 10.1038/srep24044 (PMC4820774; doi:10.1038/srep24044)
Supplement: Supplementary Information [file srep24044-s1.pdf]

Supplementary information

A four-dimensional snapshot hyperspectral video-endoscope for bio-imaging applications

Hoong-Ta Lim, Vadakke Matham Murukeshan

Centre for Optical and Laser Engineering, School of Mechanical and Aerospace Engineering, Nanyang Technological University, 50 Nanyang Avenue, Singapore 639798

Correspondence and requests for materials should be addressed to V.M.M. (email: mmurukeshan@ntu.edu.sg)

(i) Reflectance imaging using chicken breast tissue with blood clot.

In this experiment, a chicken breast tissue with a blood clot was used as the sample (Supplementary Fig. 1a) and imaged. The sample is divided into Regions B1, B2 and B3. Region B1 is the chicken breast tissue. Region B2 is a thin layer of blood clot on the chicken breast tissue. It can be observed from Supplementary Fig. 1b that the chicken breast tissue is still partially visible in Region B2. Region B3 is the blood clot. The sample was manually moved using a mechanical stage towards the right of the 2-D end of the fiber bundle during data acquisition. With respect to the sample, the 2-D end of the fiber bundle was moving downwards (arrow in Supplementary Fig. 1b). A total of 80 frames were taken at a rate of ~6.16 Hz.

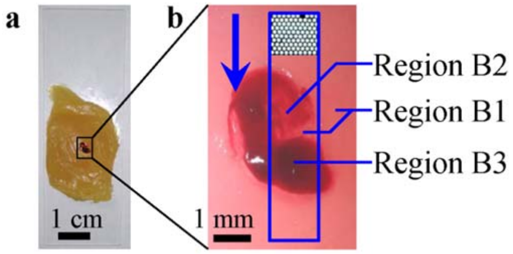

Supplementary Figure 1. (a) Sample of chicken breast tissue with blood clot for reflectance imaging and (b) the photograph of the 2-D end of the fiber bundle superimposed on the sample. Region B1 is the chicken breast tissue. Region B2 is a thin layer of blood clot on the chicken breast tissue which is still partially visible. Region B3 is the blood clot. The fiber bundle in (b) shows its initial position and the arrow indicates its relative movement with respective to the sample during data

acquisition. (a) and (b) are images of the same sample but appear to have different colors due to the different illuminations and cameras used.

Supplementary Fig. 2 shows the reflectance mappings of nine datacubes at 600 nm. Supplementary Videos 3 and 4 are made up of 80 frames each, using reflectance mappings of 450 nm and 600 nm, respectively. By looking at the frames in Supplementary Fig. 2 sequentially and Supplementary Videos 3 and 4, it can be further confirmed that the proposed system was able to perform HS reflectance imaging in a snapshot configuration. The different reflectance between Regions B1, B2 and B3 can be differentiated from each another. The 2-D end of the fiber bundle was initially imaging Region B1 of high reflectance. Then it moved downwards with respect to the sample and started to image Region B2 of moderate reflectance on its left. Following this path, it started to image Region B3 of low reflectance and proceeded to image Region B1 again before data acquisition stopped. These depict the actual relative motion between them during data acquisition (Supplementary Fig. 1b). Close to the center of the blood clot, there was a small area of Region B1 in between Regions B2 and B3. This area can be seen in Frame 33 of Supplementary Fig. 2 which represented its size and shape correctly.

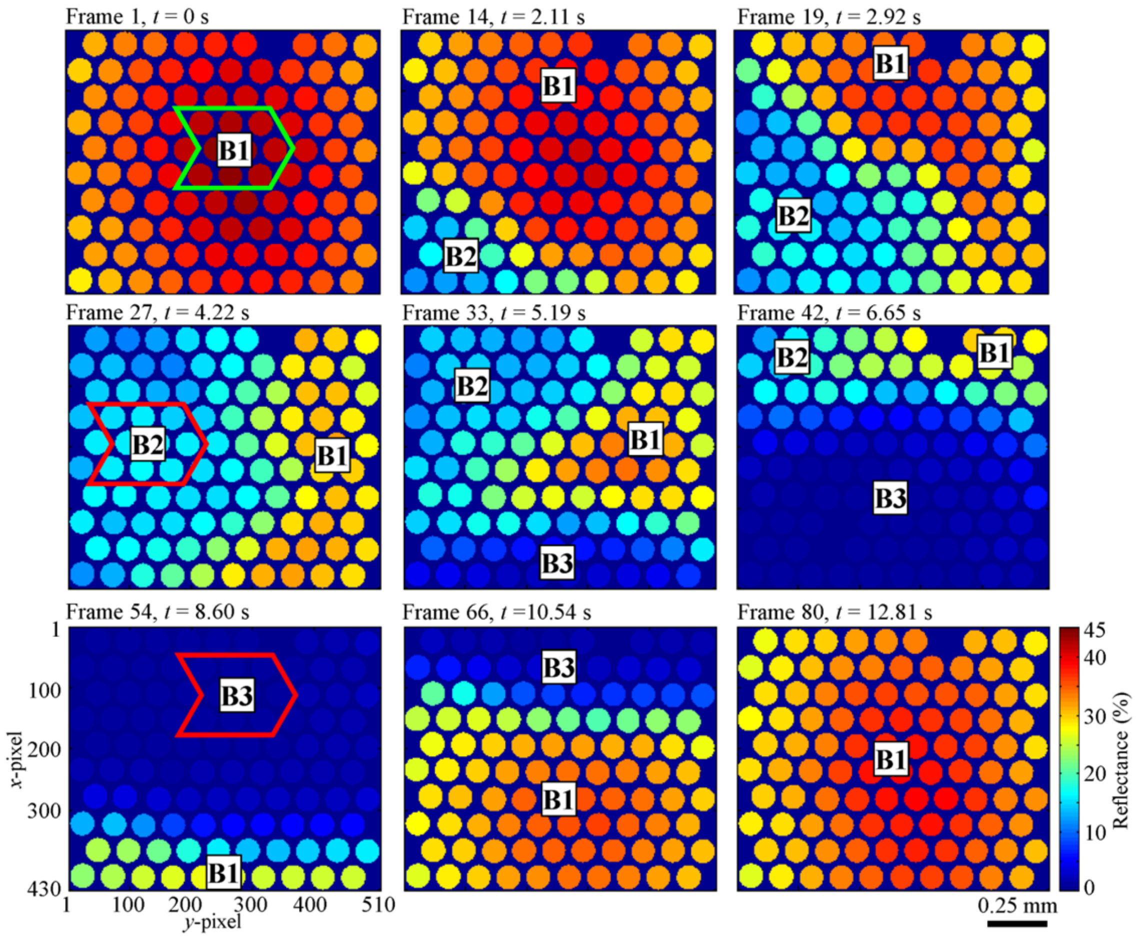

Supplementary Figure 2. Reflectance mappings of nine datacubes at 600 nm taken at different timings. The 2-D end of the fiber bundle was moving downwards with respect to the sample during data acquisition.

The mean reflectance spectra and standard deviations of Regions B1, B2 and B3 are shown in Supplementary Fig. 3. Each data set was calculated from 27 spectra. The spectra of Regions B1, B2 and B3 were acquired from 9 fiberlets whose positions are indicated by the arrow boxes in Supplementary Fig. 2, and from Frames 1-3, 26-28 and 53-55 respectively.

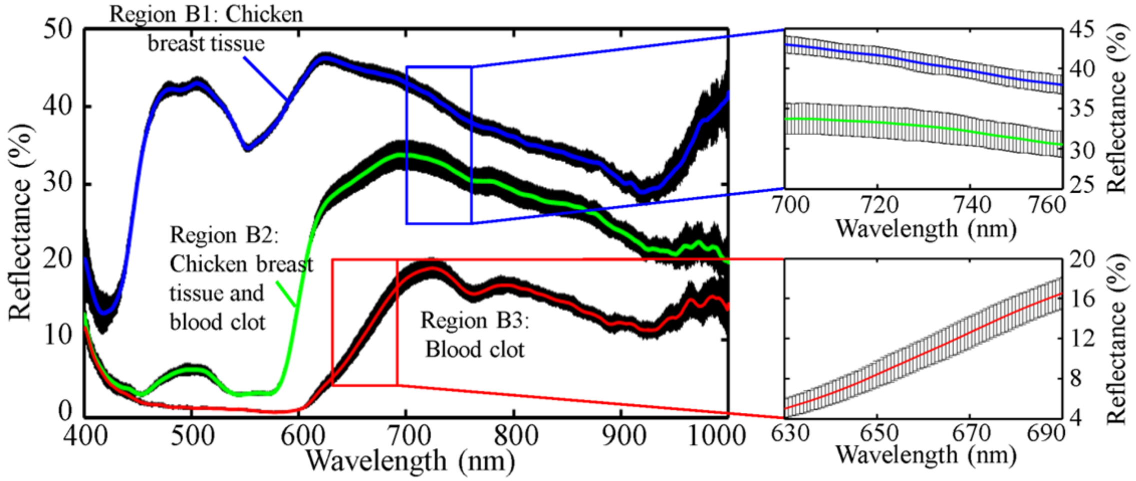

Supplementary Figure 3. Reflectance spectra of Regions B1, B2 and B3. Mean reflectance spectrum (center bold line) and standard deviation (surrounding black areas) along with insets.

The spectra in Supplementary Fig. 3 show that the 4-D HS imaging probe could capture the detailed reflectance spectra of Regions B1, B2 and B3 while there was a relative motion between the sample and the 2-D end of the fiber bundle. It can be observed that Region B1 (chicken breast tissue) had the highest reflectance, while Region B3 (blood clot) had the lowest. The reflectance spectrum of Region B2 is between the spectra of Regions B1 and B3. This could be due to Region B2 having the thin layer of blood clot causing the chicken breast tissue underneath it to be still partially visible. The average standard deviations of the reflectance spectra of Regions B1, B2 and B3 were about ±1.31%, ±1.37% and ±0.98% respectively.

(ii) Data acquisition- Details

Data acquisition was done using the dedicated software of the detector camera (SOLIS, Andor). The selected ROI was 1004×756 pixel^2 (spatial×spectral) which corresponds to the spectral range of interest from 400-1000 nm. Although the exposure time was set to 0.1 s, the software set the kinetic cycle time to 0.16221 s. Therefore the images were acquired at a rate of ~6.16 Hz. The electron-multiplying gain of the detector camera was turned off for reflectance imaging, but set to 100 for fluorescence imaging. During the experiment, the detector camera captured a series of 1004×756 pixel^2 images at a rate of ~6.16 Hz until the number of images taken matched the pre-determined number of images to capture. Each image was named in sequence and saved as separate file after the experiment.

(iii) Data processing and visualization- Details

Data processing was done offline using MATLAB®. In reflectance imaging, *Sample* data was acquired from the sample. The *Sample* data was corrected using dark reference (*Dark*) and white reference (*White*) using equation (1) to get the *Reflectance* data.

$$Reflectance\ (x, \lambda, frame) = Smooth\left[\frac{Sample\ (x,\lambda,frame) - Dark\ (x,\lambda)}{White\ (x,\lambda) - Dark\ (x,\lambda)}\right] \times 0.99. \quad (1)$$

Dark data was acquired when the broadband light source was turned off and the forelens covered. It represents the image with dark current noise where the reflectance was 0%. *White* data was acquired by imaging the 99% reflectance standard where the reflectance was 99%. A set of ten images were taken and averaged to give the *Dark* and *White* data. x and λ refer to the column and the calibrated spectral band allocated to the row of the sensor array's selected ROI respectively. *Frame* refers to the number of images taken for "sample data", and in this case it was 80. *Smooth* is the 9-point moving average in the spectral direction for spectrum smoothing.

In fluorescence imaging, *Sample* data was acquired from the sample and 160 images were taken. The *Sample* data was corrected using a dark reference (*Dark*) and the quantum efficiency of the detector camera (*QE*) using equation (2) to get the *Fluorescence* data.

$$Fluorescence\ (x, \lambda, frame) = Norm\left\{\frac{Smooth[Sample\ (x,\lambda,frame) - Dark\ (x,\lambda)]}{QE(\lambda)}\right\}. \quad (2)$$

Dark data was acquired when the laser was turned off and the forelens covered. It represents the image with dark current noise and without any fluorescence. *QE* took into account the varying sensitivities the detector camera had with different wavelengths. A set of ten images were taken and averaged to give the *Dark* data. *Norm* was to normalize the entire data set to one.

After applying the calculations, the reflectance and fluorescence measurements were having a spatial-spectral-frame data of 1004×756×*frame*. Using the spatial calibration done on the 1-D end of the fiber bundle, the spectrum for each fiberlet was extracted from the relevant spatial positions to form a fiber-spectral-frame data of 100×756×*frame*. Since Fiberlet 4 was inactive, its spectrum was assigned to be zero. Using the data from each *frame*, there was a digital reconstruction step to remap the spectrum of each fiberlet back to the respective position on the 2-D end of the fiber bundle. In order to get a correct visualization of the imaged sample, the data was flipped horizontally in the spatial direction as

the left side of the 2-D end of the fiber bundle was used to image the right side of the sample, and *vice versa*.

Supplementary Video

The relevant videos demonstrating the proposed concepts and the related data are as per below.

Supplementary Video 1. A reflectance video with 80 frames of 575 nm at ~6.16 Hz acquired during the imaging of the simulated tissue sample.

Supplementary Video 2. A reflectance video with 80 frames of 700 nm at ~6.16 Hz acquired during the imaging of the simulated tissue sample.

Supplementary Video 3. A reflectance video with 80 frames of 450 nm at ~6.16 Hz acquired during the imaging of the sample of chicken breast tissue with blood clot.

Supplementary Video 4. A reflectance video with 80 frames of 600 nm at ~6.16 Hz acquired during the imaging of the sample of chicken breast tissue with blood clot.

Supplementary Video 5. A fluorescence video showing frames 11-135 (of 160 captured frames) of 575 nm at ~6.16 Hz acquired during the imaging of the simulated tissue sample.

Supplementary Video 6. A fluorescence video showing frames 11-135 (of 160 captured frames) of 585 nm at ~6.16 Hz acquired during the imaging of the simulated tissue sample.
